# Factors affecting the clinical relevance of *Corynebacterium striatum* isolated from blood cultures

**DOI:** 10.1371/journal.pone.0199454

**Published:** 2018-06-21

**Authors:** Seung Ji Kang, Su-Mi Choi, Jin-A Choi, Jin Un Choi, Tae-Hoon Oh, Seong Eun Kim, Uh Jin Kim, Eun Jeong Won, Hee-Chang Jang, Kyung-Hwa Park, Jong Hee Shin, Sun-Seog Kweon, Sook-In Jung

**Affiliations:** 1 Department of Internal Medicine, Chonnam National University Medical School, Gwangju, South Korea; 2 Department of Laboratory Medicine, Chonnam National University Medical School, Gwangju, South Korea; 3 Department of Preventive Medicine, Chonnam National University Medical School, Gwangju, South Korea; Universidade Federal do Rio de Janeiro, BRAZIL

## Abstract

This study aimed to identify clinical or microbiological factors affecting the clinical relevance of *Corynebacterium striatum* isolated from blood cultures. A total of 64 isolates from 51 patients identified as *C*. *striatum* by matrix assisted laser desorption/ionization time-of-flight mass spectrometry (MALDI-TOF MS) and 16S rRNA gene sequencing were assessed. More than two blood cultures were positive in 25 (48.1%) patients. Diabetes, solid tumor, and a history of previous exposure to antibiotics were more common in patients with multiple positive blood cultures. Charlson comorbidity scores were also higher, and more isolates were recovered after 48 hours of hospital stay in patients with multiple positive blood cultures. Strains recovered from patients with multiple positive blood cultures produced significantly more biofilm. Based on multilocus sequence typing (MLST), sequence type (ST) 20 (31.3%) was the most dominant, followed by ST2 (20.3%) and ST23 (10.9%). There was no relationship between the number of positive blood culture sets and sequence typing. In multivariate analyses, Carlson comorbidity score (odds ratio [OR], 1.91; 95% confidence interval [CI], 1.09–3.36; *P* = 0.03) and biofilm formation were associated with multiple positive blood cultures (OR, 17.43; 95% CI, 3.71–81.91; *P* = 0.03). This study provides evidence that the biofilm phenotype could contribute to determining the clinical significance of *C*. *striatum* in patients with severe underlying conditions. The predominance of certain STs suggests the relatedness of *C*. *striatum* infection and the nosocomial environment.

## Introduction

*Corynebacterium striatum* is one of the most frequently isolated species of coryneform bacteria in clinical microbiology laboratories [1, 2]. Because *C*. *striatum* normally inhabits the skin and mucus membrane of humans [3], the isolation of this bacterium in clinical specimens is generally regarded as contamination. However, several clinical infections of *C*. *striatum*, including pneumonia, endocarditis, and septicemia, have been reported over the last decades [4–8]. Although multiple positive blood cultures have been shown to improve the positive predictive value for true bacteremia [1, 9, 10], it is difficult to clearly distinguish true infection from colonization or contamination. Patients with true bacteremia tend to be younger and have more severe underlying diseases, such as malignancy and neutropenia, compared with those with contamination [10]. In addition to a higher number of positive blood culture sets, reduced time to positivity has also been associated with clinical significance [1]. A number of studies have demonstrated the ability for biofilm production in *C*. *striatum* isolated from patients with invasive infection, suggesting that biofilm formation is a virulence factor of this organism [9, 11]. This finding is supported by the fact that most *C*. *striatum* infections are related to implanted medical devices [7, 12–14]. However, it remains unclear whether clinical infection is associated with the biofilm formation ability of *C*. *striatum*.

*C*. *striatum* has been reported as a pathogen responsible for nosocomial respiratory infection outbreaks in patients with chronic obstructive pulmonary disease [15–17]. Molecular epidemiological studies have revealed patient-to-patient transmission via the hands of healthcare personnel or the environment [18–20]. Transient carriage has only been identified among healthcare personnel, whereas the same clone could persist in the environment or colonize a patient’s airways for months [19, 20]. In addition, *C*. *striatum* with distinct pulsed-field gel electrophoresis (PFGE) patterns were repeatedly isolated from bloodstream specimens during a three-year monitoring period, suggesting that these strains possibly repeatedly infect patients or are firmly colonized in hospital environments [9]. Recent studies have uniformly reported the emergence of multi-drug resistant (MDR) *C*. *striatum* [21, 22]. In addition, the most recent genomic study of MDR *C*. *striatum* revealed its capacity for clonal spread within and across healthcare institutions [23]. These studies show that *C*. *striatum* could constitute an important issue in healthcare associated infection.

In this study, we hypothesized that clinically relevant strains of *C*. *striatum* possess certain virulence trait such as capacity of biofilm formation, comparing with contaminant. Therefore, we aimed to find factors affecting clinical significance of *C*. *striatum* isolated from blood culture by comparing clinical and microbiologic characteristics including antimicrobial susceptibility, ability of biofilm production, genotyping of strains between groups with multiple and single positive blood culture.

## Materials and methods

### Bacterial strains

A total of 300 gram-positive bacilli identified from blood using conventional methodologies were stored at -80°C and assessed in this study. Samples were collected between 2006 and 2016 at two tertiary hospitals (Chonnam National University Hospital and Chonnam National University Hwasun Hospital, Korea). Twenty-three *C*. *striatum* isolates from sites other than blood, which were considered to be colonizers, were also collected from May to August 2016.

### Identification of isolates by MALDI-TOF MS

Matrix-assisted laser desorption/ionization time-of-flight mass spectrometry (MALDI-TOF MS) analyses of all 300 gram-positive bacilli were performed using a Microflex MALDI Biotyper (Bruker Daltonics, Billerica, MA, USA), as previously described [24].

### Molecular identification

The complete 16S rRNA genes of all *C*. *striatum* bloodstream isolates identified by MALDI-TOF MS were sequenced. DNA was extracted using the Cell SV mini kit (GeneAll Biotechnology Co., Ltd., Seoul, Korea) and polymerase chain reaction (PCR) was performed in a final volume of 50 μL using the 16SF27 and 16SR1492 primers (S1 Table) [25]. Bidirectional sequencing was performed using an ABI Prism 3730xl Genetic Analyzer (Applied Biosystems, USA) [26] and sequence analyses were performed using the Basic Local Alignment Search Tool (BLAST) on the National Center for Biotechnology Information (NCBI http://blast.ncbi.nlm.nih.gov/) database and Lasergene software (DNASTAR, USA). Species identification required ≥ 99% 16S rRNA gene identity and ≥ 0.8% separation [27].

### Antimicrobial susceptibility testing

Antibiotic susceptibility testing was performed using E-test (AB BIODISK, Solna, Sweden) strips on Mueller-Hinton agar plates supplemented with 5% sheep blood (Kisanbio, Korea) [28]. The breakpoints for susceptibility were adopted from the guidelines of the Clinical and Laboratory Standard Institute [29].

### Patients and clinical data

Medical records were reviewed for all patients with *C*. *striatum* bloodstream isolates. Patients were divided into two groups according to the number of positive blood cultures (i.e., single vs. multiple). The source of bacteremia, the appropriateness of antibiotic therapies, and the treatment outcomes were also evaluated in patients with multiple positive blood cultures. The Institutional Review Boards of the two participating hospitals (Chonnam National University Hospital, CNUH-2018-082 and Chonnam National University Hwasun Hospital, CNUHH-2018-054) approved this study. A waiver of consent was granted given the retrospective nature of the project.

### Biofilm formation assay

Biofilm formation in non-duplicate isolates was evaluated on the negatively charged polystyrene surfaces of flat-bottomed 96-well microtiter plates [9, 11]. A total of 200 μL of a 10^8^ colony forming unit(CFU)/mL bacterial suspension in brain-heart infusion (BHI) broth were added to each well of the 96-well plate and incubated at 37°C for 48 h. Next, each well was washed twice with 200 μL phosphate-buffered saline (0.01 M, pH 7.2). Biofilms were fixed with 200 μL of 99% methanol and stained with 2% crystal violet. The dye was eluted with 160 μL of 95% ethanol and the optical density (OD) at 595 nm (OD_595_) was measured using an Epoch Microplate Spectrophotometer (Bio-Tek, USA). The negative controls contained only BHI. Subtractions of the mean OD_595_ of triplicates from the negative control were used for analysis.

### Multilocus sequence typing (MLST)

In addition to the 16S rRNA gene, the internal transcribed spacer 1 (ITS1), *gyr*A, and partial *rpo*B genes were also amplified and sequenced for all *C*. *striatum* bloodstream isolates [30]. Primers were used as previously described (S1 Table) [30]. Obtained sequences were aligned and compared to those available in the European Nucleotide Archive (http://www.ebi.ac.uk/ena, HE586270 to HE586300). For each gene, previously reported allele numbers were used when exact matches were found in the European Molecular Biology Laboratory (EMBL) databases; otherwise, new numbers were arbitrarily assigned [30]. Sequence types (STs) were comprised of the distinct combination of the four alleles. If the combination of alleles had previously been reported [30], the same ST was assigned; otherwise, new STs were assigned. The nucleotide diversity for each gene was determined using DnaSP v. 5.1 [31]. The number of polymorphic sites was calculated by comparing the sequences of each *C*. *striatum* isolates and HE586270 for 16S rRNA, HE586276 for ITS1, HE586288 for *gyr*A and HE586295 for partial *rpo*B as references, respectively.

### Statistical analysis

Fisher’s exact tests or Pearson χ^2^ tests were used to compare categorical variables. Continuous variables were analyzed using the Mann-Whitney U tests or paired t-tests. One-way ANOVA or the Kruskal-Wallis test were used to compare the means of three or more continuous variables. Multivariate analysis to evaluate the factors associated with multiple positive blood cultures was performed in the enter mode with selected variables because of the limited sample size [32]. All tests of significance were two-tailed and *P*-values < 0.05 were considered statistically significant. SPSS v.18.0 (SPSS, Chicago, IL, USA) and GraphPad Prism v.5.03 (GraphPad Software, San Diego, CA, USA) were used for data analyses.

## Results

Of the 300 gram-positive bacilli isolated from blood samples, 138 (46.0%) were identified to the species-level by MALDI-TOF MS. Of these, 65 (21.7%) were identified as *C*. *striatum* and were subjected to 16S rRNA sequencing; 64 (recovered from 51 patients) had ≥ 99% similarity to one of the following *C*. *striatum* strains: AY008302, JF342700, JX974430, or KJ934789. A discrepancy between MALDI-TOF MS and 16S rRNA sequencing was found in a single strain showing 99% similarity to *C*. *simulans* (AF537604), which is closely related to *C*. *striatum* based on a previous phylogenetic analysis study [33].

A total of 64 *C*. *striatum* isolates were obtained from 51 patients. More than two blood cultures were positive in 25 (48.1%) patients, while the other 26 (51.9%) patients had one positive blood culture. The prevalence of diabetes (44.0% vs. 11.5%, *P* = 0.01) and solid tumors (20.0% vs. 0%, *P* = 0.02) was higher among patients with multiple positive blood tests compared to patients with a single positive blood culture. The Charlson comorbidity scores (5.4 ± 2.9 vs. 3.4 ± 2.4, *P* = 0.01) were also higher in patients with multiple positive blood cultures. Isolates recovered after 48 h of hospital stay (96.0% vs. 69.2%, *P* = 0.02) or previous exposure to antibiotics (100% vs. 65.4%, *P* < 0.01) were more common among patients with multiple positive blood cultures than in those with a single positive blood culture (Table 1).

**Table 1:** Comparison of demographics and clinical characteristics between the multiple and single positive blood culture groups.

Characteristics	Multiple positive blood cultures (n = 25)	Single positive blood culture (n = 26)	*P* value
Age	67.4 ± 13.8	56.4 ± 24.8	0.06
Male	10 (40.0)	15 (57.7)	0.27
Strain recovered after 48 h of hospital stay	24 (96.0)	18 (69.2)	0.02
Days of hospitalization before culture was obtained (median, IQR)	16 (9–23.5)	13.0 (0–24.5)	0.35
Comorbidities			
Diabetes mellitus	11 (44.0)	3 (11.5)	0.01
Neurologic disease	5 (20.0)	4 (15.4)	0.73
Ischemic heart disease	5 (20.0)	2 (7.7)	0.25
Liver cirrhosis	2 (8.0)	1 (3.8)	0.61
Chronic kidney disease	6 (24.0)	2 (7.7)	0.14
Renal replacement therapy	3 (12.0)	1 (3.8)	0.35
Solid tumor	5 (20.0)	0 (0)	0.02
Hematologic malignancy	3 (12.0)	2 (7.7)	0.67
Neutropenia	3 (12.0)	2 (7.7)	0.67
Recent surgery (within 1 month)	7 (28.0)	6 (23.1)	0.76
Previous exposure to any antibiotics	25 (100)	17 (65.4)	< 0.01
Presence of CVC	18 (72.0)	11 (42.0)	0.05
Indwelling device other than CVC	18 (72.0)	14 (53.8)	0.25
Ventilator prior to bacteremia (≥ 48h)	10 (40.0)	6 (23.1)	0.24
CRRT prior to bacteremia (≥ 48h)	5 (20.0)	2 (7.7)	0.25
Charlson comorbidity score	5.4 ± 2.9	3.4 ± 2.4	0.01
APACHE II score^†^	24.8 ± 11.1	21.6 ± 9.0	0.47

IQR, interquartile range; CVC, central venous catheter; CRRT, continuous renal replacement therapy; APACHE II, Acute Physiology and Chronic Health Evaluation II.

† Available for 20 and 18 patients in each group, respectively.

All isolates were susceptible to vancomycin, linezolid, and daptomycin. Most strains were highly resistant to penicillin (MIC_90_ > 32), cefotaxime (MIC_90_ > 32), clindamycin (MIC_90_ > 256), erythromycin (MIC_90_ > 256), and levofloxacin (MIC_90_ > 32). There was no difference in antibiotic susceptibility between groups with multiple and single positive cultures (S2 Table).

The 16S rRNA, ITS1, *gyr*A, and *rpo*B genes had 25, 184, three, and 67 polymorphic sites, respectively, and the average number of nucleotide differences was 2.1, 13.1, 1.0, and 6.4, respectively (S3 Table). Using the four genes, 19 distinct STs were identified (ST2 and ST20–37). ST2 has been previously reported [30] and was found in 12 isolates (23.5%) in this study. Another 18 distinct STs (ST20–37) were also identified; ST20 (27.5%) was the most abundant, followed by ST2 (23.5%), 23 (9.8%), 22, 24, 25, 28 (two isolates each), and 21, 26, 27, 29, 30, 31, 32, 33, 34, 35, 36, and 37 (one isolate each; S4 Table). Twelve STs (ST2, 20, 21, 23, 24, 25, 28, 29, 31, 32, 34, and 36) were distributed among the multiple positive blood culture isolates and lineage ST20 was the most common (36.0%), while 12 STs (ST2, 20, 22, 23, 24, 26, 27, 28, 30, 33, 35, and 37) were identified in the single positive culture isolates and lineage ST2 was the most abundant (30.8%). Five STs (ST2, 20, 23, 24, and 28) were present in both multiple and single positive blood cultures. There was no relationship between the number of positive blood culture sets and sequence typing (Table 2). ST2 was the most abundant among isolates collected prior to 2010 (68.4%, 13 of 19 isolates), while ST20 was the most abundant after 2014 (48.8%, 21 of 40 isolates). However, spatial relationships were not clearly determined.

**Table 2:** Biofilm formation (OD_595_) by 51 non-duplicate bloodstream isolates of *C*. *striatum* according to sequence type (ST).

ST	Total isolates		Multiple positive blood cultures (n = 25)		Single positive blood culture (n = 26)	
	No.	Biofilm^b^ (mean ± SD)	No.	Biofilm (mean ± SD)	No.	Biofilm (mean ± SD)
20	14	1.82 ± 0.84	9	2.18 ± 0.79	5	1.17 ± 0.48
2	12	2.23 ± 0.88	4	3.23 ± 0.38	8	1.73 ± 0.54^c^
23	5	1.92 ± 0.85	2	2.77 ± 0.02	3	1.35 ± 0.48
Others^a^	20	1.99 ± 0.73	10	2.43 ± 0.62	10	1.56 ± 0.57^c^
Total	51	2.00 ± 0.80	25	2.50 ± 0.71	26	1.51 ± 0.54^c^

^a^Includes: ST22, ST24, ST25, and ST28 (two isolates each) and ST21, ST26, ST27, ST29, ST30, ST31, ST32, ST33, ST34, ST35, ST36, and ST37 (one isolate each).

^b^OD at 595 nm; biofilm-forming ability was determined using the crystal violet binding assay.

^c^*P* < 0.05, significant difference in biofilm-forming ability between multiple positive and single positive blood cultures.

**Fig 1:**
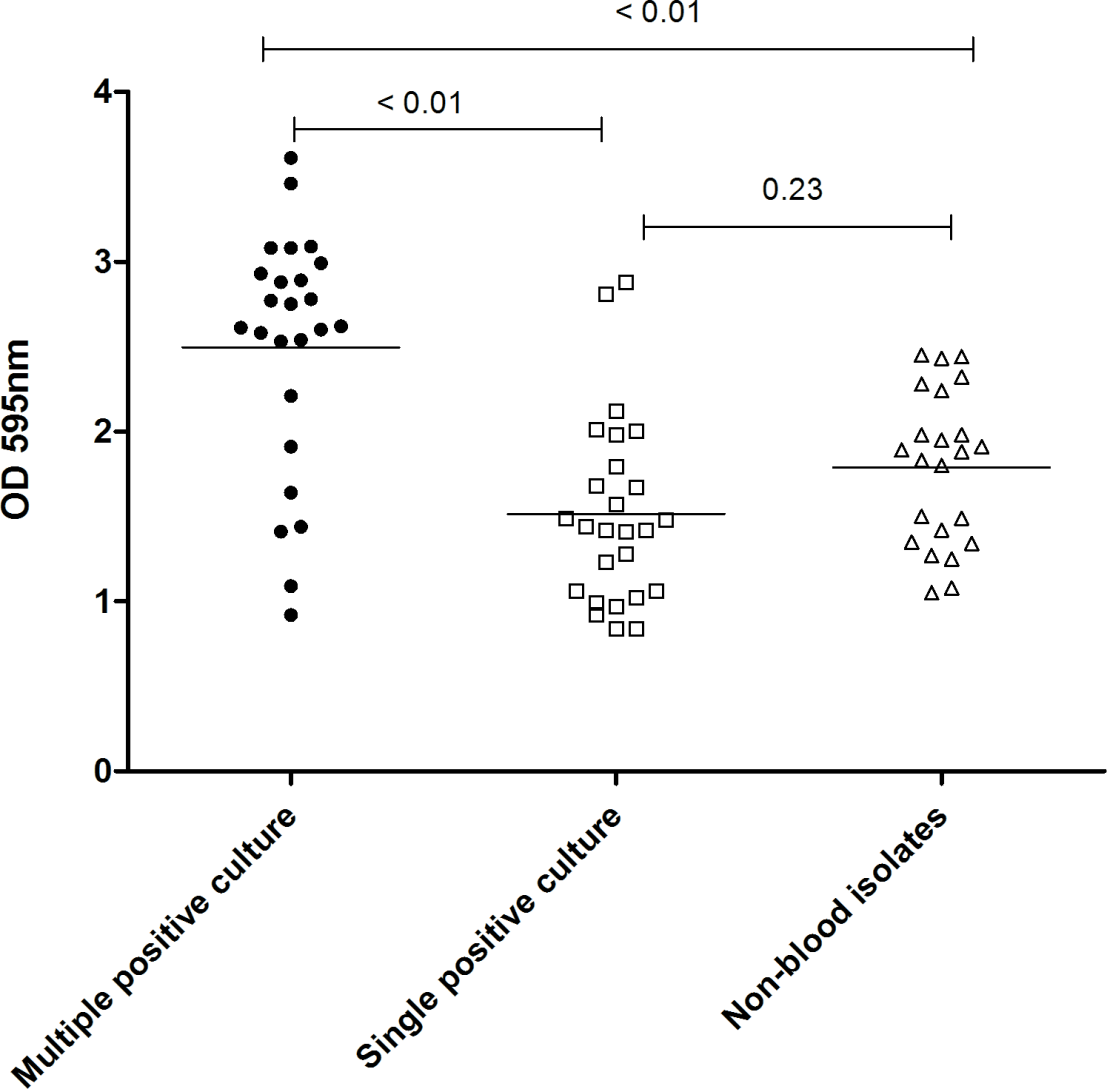
Comparison of biofilm formation. Following 48 h incubation on a polystyrene 96-well plate surface in brain-heart infusion broth, the average absorbance (OD_595_) obtained with the crystal violet assay was evaluated and compared between strains from multiple positive blood cultures (n = 25), single positive blood culture (n = 26), and non-blood isolates (n = 23). Non-blood samples were obtained from peritoneal fluid (n = 2), peritoneal dialysate (n = 1), ear discharge (n = 2), pleural fluid (n = 1), urine (n = 6), endotracheal aspiration (n = 1), and open wounds (n = 10). One-way ANOVA with post-hoc analysis was used to compare the means of three continuous variables.

*C*. *striatum* isolates from patients with multiple positive blood cultures produced more biofilm compared to isolates from patients with single positive blood cultures or non-blood specimens (*P* < 0.05; Fig 1). No statistical differences in biofilm formation were observed based on ST (Table 2). Notably, both multiple and single positive blood culture isolates belonging to ST2 were the greatest biofilm producers. Among these ST2 isolates, those from multiple positive blood cultures produced more biofilm than those from single positive blood cultures (OD_595,_ 3.23 ± 0.38 vs. 1.73 ± 0.54; *P* = 0.01).

Multivariate analysis was performed to determine factors associated with multiple positive blood cultures. Because of the limited sample size, selected variables–age, Charlson comorbidity score, presence of central venous catheter (CVC), and biofilm production–were included in the analysis. Charlson comorbidity score (odds ratio [OR], 1.91; 95% confidence interval [CI], 1.09–3.36; *P* = 0.03) and biofilm formation (OR, 17.43; 95% CI, 3.71–81.91; *P* = 0.03) were independently associated with multiple positive blood cultures (Table 3).

**Table 3:** Predictive factors for multiple positive blood cultures of *C*. *striatum* by multiple logistic regression analysis.

Variable	Category	Adjusted OR (95% CI)	*P* value
Age (year)		0.99 (0.92–1.04)	0.82
Carlson comorbidity score		1.91 (1.09–3.36)	0.03
Presence of CVC	No	1.00	
	Yes	6.61 (0.92–47.60)	0.06
Biofilm formation		17.43 (3.71–81.91)	< 0.01

OR, odds ratio; CI, confidence interval; CVC, Central venous catheter.

## Discussion

Since MALDI-TOF MS was introduced to our clinical lab in October 2014, detailed identification of gram-positive bacilli has been performed. Over two years, 87,241 blood cultures were submitted and 713 gram-positive bacilli were recovered. Among these strains, *C*. *striatum* was the most frequently identified species (84 isolates, 11.8%), consistent with other studies [1, 2]. Given the prevalence and increasing clinical significance of *C*. *striatum*, this species warrants greater attention among gram-positive bacilli [1, 4–8]. However, sufficient data are not available to guide physicians in the interpretation of positive *C*. *striatum* blood cultures. Therefore, this study was performed to identify a predictive factor for clinical relevance using 64 *C*. *striatum* bloodstream isolates recovered from 51 patients between 2006 and 2016. To the best of our knowledge, this is the first study to investigate the microbiological and clinical characteristics, as well as the genetic relatedness, of bloodstream isolates in a longitudinal manner.

The number of positive blood cultures is one proven methodology that can help differentiate true infection from contamination [34]. Thus, we divided the patients according to the number of positive blood cultures. Patients with multiple positive blood cultures had more underlying illnesses, including diabetes, solid tumors, higher Charlson comorbidity scores, and increased exposure to antibiotics, than those with single positive blood cultures. The most common source of bacteremia in patients with multiple positive blood cultures was catheter related. These clinical findings are consistent with previous studies that reported that most invasive *C*. *striatum* infections were related to underlying medical conditions including exposure to broad spectrum antibiotics, immunocompromised hosts, invasive medical procedures, and implanted medical devices [4–7]. However, to date, few studies have investigated the microbiological characteristics and virulence factors of *C*. *striatum* as related to invasive infection.

In the present study, in order to investigate whether distinct microbiological characteristics are related to the clinical relevance of *C*. *striatum* blood isolates, we tested antibiotic susceptibility, genotyping using MLST, and biofilm production ability. To the best of our knowledge, no single marker robust enough to be of clinical value has been identified. *Staphylococcus epidermidis*, which is similar to *C*. *striatum*, is a common inhabitant of the skin and mucous membranes and also causes opportunistic infection related to medical devices. *S*. *epidermidis* isolates causing catheter related bloodstream infections have been shown to be multidrug resistant, *icaA* positive (which involves biofilm formation), and belong to specific PFGE or MLST types, compared to those from colonized healthcare workers [35]. A previous study demonstrated that the ITS1, *gyr*A, and *rpo*B genes possess adequate variability for discriminating between the MLST types of *C*. *striatum* strains [30]. However, in contrast to a previous study that described the 16S rRNA gene as highly conservative, we identified five distinct 16S rRNA sequences with 25 polymorphic sites. Baio *et al*. also assessed the 16S rRNA gene and observed variability via phylogenetic analyses [26]. Based on these four housekeeping genes, there was no difference in ST distribution between the multiple and single blood culture groups. Interestingly, among the 19 distinct STs generated in the current study, > 60% of both multiple and single positive blood culture isolates shared five STs: ST2, 20, 23, 24, and 28. Of these, ST20 and ST2 were dominant in both groups. However, there was no direct spatial/temporal relationship within the same STs; 82% of the *C*. *striatum* isolates were recovered after 48 hours of hospital stay in the current study. As there is no standard MLST of *C*. *striatum* and only a limited number of studies have addressed this issue, it remains somewhat unclear which strains are commonly circulating in Korea or other areas. However, as ST2 was also the most frequently observed ST in previous studies, it is possible that ST2 is the most common strain circulating in hospital settings [20, 30]. Furthermore, certain strains has been predominantly isolated in a non-outbreak setting [23]. These findings suggest the possibility that some endemic strains persist within a nosocomial environment, although it is unclear whether *C*. *striatum* isolates in the single positive blood culture group originated from healthcare workers or patients. In the present study, *C*. *striatum* biofilm production was higher in strains isolated from the multiple positive blood culture group compared to the non-blood culture controls or isolates from patients with single positive blood cultures. Even within the same ST, a higher level of biofilm production was observed in strains from multiple positive culture patients compared to the single positive culture group. The difference in biofilm formation was not associated with ST, consistent with a previous study by Qin *et al*. showing no relationship between PFGE type and level of biofilm formation [9]. It is possible that some *C*. *striatum* strains may have evolved the ability to form robust biofilms under specific circumstances. This hypothesis might be supported by previous studies demonstrating that the increased level of biofilm formation was associated with the infection status and not strain clonality in *Staphylococcus aureus* and Enterococcal disease [36, 37]. In contrast to our finding, Souza *et al*. showed that biofilm production in *C*. *striatum* was related to PFGE type [11]. Considering the different methodologies used for strain typing and biofilm formation measurement, further investigations should be conducted to establish the relatedness between strain type and biofilm production.

Most isolates tested in this study were resistant to penicillin, cefotaxime, clindamycin, erythromycin, and levofloxacin. This is in agreement with recent studies that have shown that clinical *C*. *striatum* strains are multidrug resistant. As earlier studies have described broad sensitivity to various antibiotics [2, 38], while more recent publications have reported multidrug resistance [8, 26], it is assumed that resistance has been acquired by adaptation to antibiotic selection. In this context, most patients in this study were exposed to multiple antibiotics before the *C*. *striatum* strains were isolated. An emerging resistance to daptomycin has been reported in several studies [13, 39, 40]; however, no such resistance was observed in this study. Most isolates were collected prior to the introduction of daptomycin in Korea and therefore, further surveillance for emerging resistance should be undertaken. Baio *et al*. reported that the multidrug resistance phenotype of *C*. *striatum* is associated with specific PFGE types [26]. However, recent studies showed that the resistance phenotype was not related to specific clones, as the majority of *C*. *striatum* isolates were multidrug resistant, which is consistent with our finding [21, 22].

This study had several limitations. First, it was a retrospective study and not all *C*. *striatum* blood isolates were collected during the study period. Thus, there could have been selection bias, despite random sample collection. Second, as patients were divided based on the number of positive blood cultures, there is a possibility that contamination could have caused some patients to have multiple positive tests. However, we regarded this classification as relatively objective based on previous findings demonstrating the importance of the number of positive blood cultures for interpreting the clinical significance of *C*. *striatum* [1, 9, 10]. Despite these limitations, this study included a large number of patients over 10 years and simultaneously evaluated the clinical and microbiological aspects of *C*. *striatum* isolates. Thus, our results may help physicians understand the clinical relevance of *C*. *striatum* and aid in managing infections more appropriately.

## Conclusions

Patients with severe underlying diseases were more likely to develop clinically significant *C*. *striatum* bacteremia. *C*. *striatum* isolates recovered from patients with multiple positive blood cultures had a significantly higher level of biofilm production, suggesting that biofilms are an important virulence factor. The predominance of specific STs during specific timeframes suggests the relatedness of *C*. *striatum* infections and the nosocomial environment. Clinicians should be aware of the possibility of invasive *C*. *striatum* infections in patients with severe underlying diseases and biofilm related infections. Timely, appropriate treatments and the implementation of strict infection control measures to prevent the spread of *C*. *striatum* are warranted.

## Supporting information

S1 Table Primers used for performing the molecular analysis of 64 *C*. *striatum* bloodstream isolates. (DOCX)

S2 Table In vitro antimicrobial susceptibility of 64 C. striatum bloodstream isolates using E-test. (DOCX)

S3 Table Genetic diversity of the 16S rRNA, ITS1, gyrA and rpoB gene among 64 C. striatum bloodstream isolates. (DOCX)

S4 Table List of 64 C. striatum blood isolates analyzed by molecular methods with location, date of isolation and biofilm formation. (DOCX)
