# Supplementary material for: Factors affecting the clinical relevance of Corynebacterium striatum isolated from blood cultures
Source: PLoS One. 2018 Jun 21;13(6):e0199454. doi: 10.1371/journal.pone.0199454 (PMC6013186; doi:10.1371/journal.pone.0199454)
Supplement: S1 Table — (DOCX) [file pone.0199454.s001.docx]

**S1 Table. Primers used for performing the molecular analysis of 64 *C. striatum* bloodstream isolates.**

| **Gene** | **Primer** | **Sequence (5’-> 3’)** | **Function** |
| --- | --- | --- | --- |
| 16S rRNA | 16SF27 | AGAGTTTGATCMTGGCTCAG | 16SrRNA |
|  | 16SR1492 | TACGGYTACCTTGTTACGACTT |  |
| *rpo*B | C2700F | CGWATGAACATYGGBCAGGT | β-subunit of RNA polymerase enzyme |
|  | C3130R | TCCATYTCRCCRAARCGCTG |  |
| ITS1 | 16F945 | GGGCCCGCACAAGCGGTGG | Interspacer sequence region 1 |
|  | 23R458 | CTTTCCCTCACGGTAC |  |
|  | rrn16SF | GAAGTCGTAACAAGG |  |
| *gyr*A | gyrA1F | GCGGCTACGTAAAGTCC | Gyrase |
|  | gyrA2R | CCGCCGGAGCCGTTCAT |  |
