# Supplementary material for: Factors affecting the clinical relevance of Corynebacterium striatum isolated from blood cultures
Source: PLoS One. 2018 Jun 21;13(6):e0199454. doi: 10.1371/journal.pone.0199454 (PMC6013186; doi:10.1371/journal.pone.0199454)
Supplement: S2 Table — (DOCX) [file pone.0199454.s002.docx]

| **Antimicrobial agents** | **MIC Range (μg/ml)** | **MIC_50_** | **MIC_90_** | **No. (%) of isolates** | | |
| --- | --- | --- | --- | --- | --- | --- |
|  |  |  |  | **R^†^** | **I^†^** | **S^†^** |
| Penicillin | 0.02~32 | > 32 | > 32 | 60 (93.8) | 3 (4.7) | 1 (1.6) |
| Cefotaxime | 0.38~32 | > 32 | > 32 | 62 (96.9) |  | 2 (3.1) |
| Clindamycin | 0.06~256 | > 256 | > 256 | 63 (98.4) |  | 1 (1.6) |
| Erythromycin | 0.02~256 | 8 | > 256 | 63 (98.4) |  | 1 (1.6) |
| Gentamicin | 0.05~16 | 3 | 12 | 3 (4.7) | 17 (26.6) | 44 (68.8) |
| Levofloxacin | 8.0~32 | > 32 | > 32 | - | - | - |
| Vancomycin | 0.38~0.75 | 0.50 | 0.75 |  |  | 64 (100) |
| Linezolid | 0.13~0.50 | 0.19 | 0.25 |  |  | 64 (100) |
| Daptomycin | 0.05~0.13 | 0.09 | 0.13 |  |  | 64 (100) |

**S2 Table. In vitro antimicrobial susceptibility of 64 *C. striatum* bloodstream isolates using E-test.**

† Clinical and Laboratory Standard Institutes interpretive criteria applied (29).
