# Supplementary material for: Factors affecting the clinical relevance of Corynebacterium striatum isolated from blood cultures
Source: PLoS One. 2018 Jun 21;13(6):e0199454. doi: 10.1371/journal.pone.0199454 (PMC6013186; doi:10.1371/journal.pone.0199454)
Supplement: S3 Table — (DOCX) [file pone.0199454.s003.docx]

**S3 Table. Genetic diversity of the 16S rRNA, ITS1, *gyr*A and *rpo*B gene among 64 *C. striatum* bloodstream isolates.**

| Locus | Fragment length (bp) | No. of alleles | No. of polymorphic site | Average No. of nucleotide difference |  |
| --- | --- | --- | --- | --- | --- |
| 16S rRNA | 1246 | 5 | 25 | 2.1 | |
| ITS1 | 350 | 5 | 184 | 13.1 |  |
| *gyr*A | 211 | 3 | 3 | 1.0 |  |
| *rpo*B | 378 | 5 | 67 | 6.4 |  |
