# Supplementary material for: Factors affecting the clinical relevance of Corynebacterium striatum isolated from blood cultures
Source: PLoS One. 2018 Jun 21;13(6):e0199454. doi: 10.1371/journal.pone.0199454 (PMC6013186; doi:10.1371/journal.pone.0199454)
Supplement: S4 Table — (DOCX) [file pone.0199454.s004.docx]

**S4 Table. List of 64 *C. striatum* blood isolates analyzed by molecular methods with location, date of isolation and biofilm formation.**

| Isolate^†^ | Culture date  (MM/DD/YY) | Ward-room | Hospital | ST | 16Sr RNA | ITS1 | gyrA | *rpo*B | Multiple positive  blood culture | Biofilm  OD 595 nm |
| --- | --- | --- | --- | --- | --- | --- | --- | --- | --- | --- |
| 1 | 12/21/07 | MICU | A | 2 | 1 | 6 | 2 | 2 | No | 2.88 |
| 2-a | 01/09/10 | 807-33 | A | 2 | 1 | 6 | 2 | 2 | Yes | 3.08 |
| 2-b | 01/15/10 | 807-33 | A | 2 | 1 | 6 | 2 | 2 | Yes | 3.29 |
| 3 | 02/11/10 | ER | B | 2 | 1 | 6 | 2 | 2 | No | 1.98 |
| 4 | 05/14/10 | ER | A | 2 | 1 | 6 | 2 | 2 | No | 1.57 |
| 5 | 06/18/10 | NSICU | A | 2 | 1 | 6 | 2 | 2 | No | 2.00 |
| 6 | 08/09/10 | ER | A | 2 | 1 | 6 | 2 | 2 | No | 1.49 |
| 7 | 08/27/10 | MICU | A | 2 | 1 | 6 | 2 | 2 | Yes | 3.61 |
| 8 | 09/26/10 | 805-62 | A | 2 | 1 | 6 | 2 | 2 | Yes | 3.46 |
| 9 | 10/12/10 | SICU | A | 2 | 1 | 6 | 2 | 2 | No | 1.44 |
| 10 | 11/03/10 | MICU | A | 2 | 1 | 6 | 2 | 2 | Yes | 2.77 |
| 11 | 11/19/10 | ER | A | 2 | 1 | 6 | 2 | 2 | No | 1.23 |
| 12 | 12/20/10 | MICU | A | 2 | 1 | 6 | 2 | 2 | No | 1.28 |
| 13 | 08/20/14 | 73-22 | B | 20 | 6 | 6 | 3 | 4 | No | 2.01 |
| 14 | 11/28/14 | SICU | A | 20 | 6 | 6 | 3 | 4 | No | 0.92 |
| 15 | 11/29/14 | 9A-63 | A | 20 | 6 | 6 | 3 | 4 | Yes | 2.21 |
| 16 | 02/10/15 | SICU | A | 20 | 6 | 6 | 3 | 4 | Yes | 1.09 |
| 17-a | 03/01/15 | SICU | A | 20 | 6 | 6 | 3 | 4 | Yes | 2.62 |
| 17-b | 03/03/15 | SICU | A | 20 | 6 | 6 | 3 | 4 | Yes | 2.56 |
| 18-a | 03/29/15 | 73-56 | B | 20 | 6 | 6 | 3 | 4 | Yes | 2.54 |
| 18-b | 03/29/15 | 73-56 | B | 20 | 6 | 6 | 3 | 4 | Yes | 3.04 |
| 18-c | 03/29/15 | 73-56 | B | 20 | 6 | 6 | 3 | 4 | Yes | 3.16 |
| 19-a | 05/20/15 | MICU | B | 20 | 6 | 6 | 3 | 4 | Yes | 3.08 |
| 19-b | 05/22/15 | MICU | B | 20 | 6 | 6 | 3 | 4 | Yes | 3.42 |
| 20 | 06/21/15 | 52-61 | B | 20 | 6 | 6 | 3 | 4 | Yes | 0.92 |
| 21 | 07/10/15 | MICU | B | 20 | 6 | 6 | 3 | 4 | Yes | 2.60 |
| 22 | 07/18/15 | 8B-28 | A | 20 | 6 | 6 | 3 | 4 | No | 0.84 |
| 23 | 07/30/15 | MICU | A | 20 | 6 | 6 | 3 | 4 | No | 1.02 |
| 24 | 07/31/15 | MICU | B | 20 | 6 | 6 | 3 | 4 | Yes | 1.64 |
| 25 | 08/21/15 | SICU | A | 20 | 6 | 6 | 3 | 4 | No | 1.06 |
| 26-a | 12/09/15 | SICU | B | 20 | 6 | 6 | 3 | 4 | Yes | 2.99 |
| 26-b | 12/09/15 | SICU | B | 20 | 6 | 6 | 3 | 4 | Yes | 3.02 |
| 26-c | 12/09/15 | SICU | B | 20 | 6 | 6 | 3 | 4 | Yes | 3.31 |
| 27 | 03/18/15 | EICU | A | 21 | 6 | 6 | 3 | 8 | Yes | 1.41 |
| 28 | 04/18/15 | NICU | A | 22 | 6 | 9 | 3 | 4 | No | 1.68 |
| 29 | 10/16/15 | ER | A | 22 | 6 | 9 | 3 | 4 | No | 1.42 |
| 30 | 07/31/10 | SICU | A | 23 | 6 | 7 | 3 | 4 | No | 1.79 |
| 31 | 07/10/15 | 8A-60 | A | 23 | 6 | 7 | 3 | 4 | No | 0.84 |
| 32-a | 08/26/15 | 808-66 | A | 23 | 6 | 7 | 3 | 4 | Yes | 2.75 |
| 32-b | 09/09/15 | 808-66 | A | 23 | 6 | 7 | 3 | 4 | Yes | 2.61 |
| 33 | 12/11/15 | EICU | A | 23 | 6 | 7 | 3 | 4 | No | 1.42 |
| 34-a | 03/08/16 | ER | A | 23 | 6 | 7 | 3 | 4 | Yes | 2.78 |
| 34-b | 03/08/16 | ER | A | 23 | 6 | 7 | 3 | 4 | Yes | 2.80 |
| 35 | 08/14/15 | ER | A | 24 | 6 | 6 | 2 | 4 | No | 1.06 |
| 36 | 12/11/15 | 808-67 | A | 24 | 6 | 6 | 2 | 4 | Yes | 2.53 |
| 37 | 11/13/14 | 8A-61 | A | 25 | 6 | 3 | 3 | 4 | Yes | 2.61 |
| 38 | 11/09/15 | CCU | A | 25 | 6 | 3 | 3 | 4 | Yes | 2.58 |
| 39 | 12/01/15 | MICU | A | 26 | 6 | 7 | 2 | 4 | No | 1.41 |
| 40 | 12/05/14 | 808-22 | A | 27 | 7 | 6 | 2 | 4 | No | 0.97 |
| 41 | 10/31/09 | SICU | A | 28 | 1 | 7 | 2 | 2 | Yes | 1.91 |
| 42 | 05/19/10 | ER | A | 28 | 1 | 7 | 2 | 2 | No | 1.48 |
| 43-a | 02/23/12 | SICU | A | 29 | 1 | 1 | 2 | 2 | Yes | 2.89 |
| 43-b | 02/24/12 | SICU | A | 29 | 1 | 1 | 2 | 2 | Yes | 3.02 |
| 43-c | 06/10/12 | SICU | A | 29 | 1 | 1 | 2 | 2 | Yes | 2.97 |
| 44 | 08/11/13 | EICU | A | 30 | 1 | 6 | 2 | 4 | No | 2.81 |
| 45 | 12/17/15 | 53-22 | B | 31 | 6 | 7 | 3 | 2 | Yes | 3.09 |
| 46-a | 06/07/06 | EICU | A | 32 | 1 | 6 | 3 | 2 | Yes | 2.93 |
| 46-b | 06/07/06 | EICU | A | 32 | 1 | 6 | 3 | 2 | Yes | 2.95 |
| 47 | 04/03/07 | ER | A | 33 | 9 | 1 | 1 | 4 | No | 1.67 |
| 48 | 11/07/15 | 73-55 | B | 34 | 6 | 1 | 3 | 4 | Yes | 1.44 |
| 49 | 02/15/16 | CCU | A | 35 | 8 | 1 | 3 | 9 | No | 2.12 |
| 50-a | 03/10/16 | 808-22 | A | 36 | 6 | 6 | 3 | 10 | Yes | 2.88 |
| 50-b | 03/10/16 | 808-22 | A | 36 | 6 | 6 | 3 | 10 | Yes | 3.39 |
| 51 | 03/24/16 | 808-62 | A | 37 | 6 | 1 | 3 | 11 | No | 0.99 |

†, -a, -b, -c represent multiple isolates collected on different days or different set of blood culture bottles on same day from the same patient

ST, sequence type; MICU, medical intensive care unit; ER, emergency room; NSICU, neurosurgical intensive care unit; SICU, surgical intensive care unit; CCU, coronary care unit; EICU, emergency intensive care unit; NICU, neurointensive care unit
